# Chytridiomycosis of Marine Diatoms—The Role of Stress Physiology and Resistance in Parasite-Host Recognition and Accumulation of Defense Molecules

**DOI:** 10.3390/md15020026

**Published:** 2017-01-25

**Authors:** Bettina Scholz, Frithjof C. Küpper, Wim Vyverman, Halldór G. Ólafsson, Ulf Karsten

**Affiliations:** 1 BioPol ehf., Einbúastig 2, 545 Skagaströnd, Iceland; halldor@biopol.is; 2 Faculty of Natural Resource Sciences, University of Akureyri, Borgir v. Nordurslod, IS 600 Akureyri, Iceland; 3 Oceanlab, University of Aberdeen, Main Street, Newburgh AB41 6AA, Scotland, UK; fkuepper@abdn.ac.uk; 4 Department of Biology, Section of Protistology and Aquatic Ecology, University of Ghent, Krijgslaan 281 S8, 9000 Ghent, Belgium; wim.vyverman@ugent.be; 5 Institute of Biological Sciences, Applied Ecology & Phycology, University of Rostock, Albert-Einstein-Strasse 3, 18059 Rostock, Germany; ulf.karsten@uni-rostock.de

**Keywords:** aldehydes, chemotaxis, chytrids, carbohydrates, amino acids, fatty acids, parasite-host interactions, phytochemicals, polyunsaturated fatty acids, stress physiology, zoospores

## Abstract

Little is known about the role of chemotaxis in the location and attachment of chytrid zoospores to potential diatom hosts. Hypothesizing that environmental stress parameters affect parasite-host recognition, four chytrid-diatom tandem cultures (*Chytridium* sp./*Navicula* sp., *Rhizophydium* type I/*Nitzschia* sp., *Rhizophydium* type IIa/*Rhizosolenia* sp., *Rhizophydium* type IIb/*Chaetoceros* sp.) were used to test the chemotaxis of chytrid zoospores and the presence of potential defense molecules in a non-contact-co-culturing approach. As potential triggers in the chemotaxis experiments, standards of eight carbohydrates, six amino acids, five fatty acids, and three compounds known as compatible solutes were used in individual and mixed solutions, respectively. In all tested cases, the whole-cell extracts of the light-stressed (continuous light exposure combined with 6 h UV radiation) hosts attracted the highest numbers of zoospores (86%), followed by the combined carbohydrate standard solution (76%), while all other compounds acted as weak triggers only. The results of the phytochemical screening, using biomass and supernatant extracts of susceptible and resistant host-diatom cultures, indicated in most of the tested extracts the presence of polyunsaturated fatty acids, phenols, and aldehydes, whereas the bioactivity screenings showed that the zoospores of the chytrid parasites were only significantly affected by the ethanolic supernatant extract of the resistant hosts.

## 1. Introduction

Parasitism is recognized as the most common consumer-resource interaction on earth and has evolved in virtually all branches of the tree of life [1]. The past two decades have seen an increasing number of emerging infectious diseases in natural and artificial ecosystems [2]. Particularly, chytridiomycosis (referred to as infections by chytrids in the following) of planktonic microalgae and cyanobacteria in freshwater ecosystems have been studied extensively during the past decades [3], suggesting that such parasites control host evolution and diversity, host community and food-web dynamics, biogeochemical cycling and the functioning of ecosystems [4]. Conversely, chytrid infections of marine microalgae and cyanobacteria and in particular diatoms (Bacillariophyceae), have only been considered in recent years (e.g., [5,6,7,8]). Although latest molecular surveys have shown that chytrids are ubiquitous and common in marine environments around the globe (e.g., [9,10,11,12]), the biology and eco-evolutionary role of marine aquatic parasites in general, and chytrids in particular remain largely unstudied.

Chytrids are true fungi and are characterized by chitinaceous cell walls [13]. Parasitic forms begin their life cycle with the attachment of a single, uniflagellate motile zoospore to the surface of an algal host cell. After zoospore attachment to a host cell, following encystment and germination, the pathogen injects its cytoplasm into the host cytoplasm, which then (in some cases, with intermediate steps) develops entirely into a parasite sporangium. When newly formed zoospores are released and infect another host cell, the infection cycle starts again [14]. In the case of the freshwater diatom *Asterionella formosa* it was shown that the parasite (*Zygorhizidium affluens* and Z. *planktonicum*) penetrates the silica shell of a host cell through the girdle region between the theca, utilizing a germ tube which grows over the surface of the diatom frustule and penetrates the host cell by squeezing between the upper and lower girdle lamellae [15].

The mechanism behind the different stages of zoospore-host recognition, ranging from the first host localization to specific signaling interactions at the host’s surface, is almost unknown. Chytrid zoospores generally swim erratically, leaping and gliding through the water, and hence swimming speed is highly variable and species specific [16]. Zoospores may be motile from a few minutes to 2–3 days only [17], but zoospores of *Rhizophydium decipiens* remained even motile inside a sporangium for 108 h [16]. Considering an average swimming speed of 160 μm·s^−1^, zoospores are able to search over considerable distances within their lifetime for suitable prey [17,18]. Most zoospores are believed to be chemotactic, that is, they respond to a chemical cue (or gradient) that guides them towards potential substrata/hosts [19]. While the uptake of external organic nutrients occurs only after germination and development of a germ-tube within prey (host) cells, the motile stages of these parasites rely on their internal storage products [20]. In the case of chytrid zoospores, it is thought that excretion products of diatoms, such as those related to photosynthesis, trigger parasite-host recognition [21]. It has been shown that zoospores of the marine chytrid *Rhizophydium littoreum* exhibit positive concentration-dependent chemotactic responses, which are elicited by carbohydrates and polysaccharides in the medium [22].

During the attachment of the zoospore to the host cell wall, at least two different responses of the hosts are distinguishable: (a) the host is susceptible to the parasite, in which case zoospore encystment and development of a sporangium will follow upon attachment of the zoospore (compatible interaction); or (b) the alga is resistant (incompatible interaction, with no observable response by the zoospore to the host, e.g., no germination). In the context of the latter response, active chemical defense of the host against the attack by a pathogen has been assumed. In fact, it has been shown that marine diatoms have evolved a variety of defensive mechanisms against grazers [23,24], including activated defenses, which involve the rapid conversion of defensive precursors into harmful molecules following cell damage (e.g., [25,26]).

Diatoms (Bacillariophyceae) are among the most ubiquitous and diverse of photosynthetic algal groups and contribute about 20%–25% of total global carbon fixation (e.g., [27,28]). Depending on seasons and geographical area, diatoms can be conspicuously abundant and they are at the basis of many pelagic and benthic food webs in aquatic ecosystems [29]. Diatoms have received particular attention in science due to their high physiological plasticity and flexibility, for example, in terms of photosynthesis [30]. In several studies, it has been shown that diatoms are able to cope with fluctuating environmental conditions by adjusting their physiology (e.g., fatty acid and/or carbohydrate compositions) (e.g., [31,32,33,34,35]). Particularly salinity influences diatom physiology directly by exerting osmotic stress. To counteract the negative effects of osmotic stress on metabolism, algae accumulate organic osmolytes that additionally also act as compatible solutes [36,37,38,39,40,41,42,43]. Compatible solutes are highly soluble, low molecular weight organic molecules without net charge at physiological pH (e.g., [43,44,45]). In algae, the osmoprotectants are restricted to four major classes of solutes: sugars and polyols, free amino acids and derivatives, quaternary ammonium compounds (e.g., homarine), and tertiary sulfonium compounds [44,45]. Among the compatible solutes, proline appears to be the most widely distributed osmolyte accumulated under environmental stress because of its high molar solubility [31,32,44,45]. Furthermore, it has been demonstrated that overall physiological responses of benthic marine diatoms to light stress such as ultraviolet radiation (UVR)-short- and long-term exposure include the accumulation of proline as well as an increase in total carbohydrates and lipids, whereas significant differences in the composition of carbohydrates, amino and fatty acids occur only after long-term exposure to UV-treatments [35].

In the present study, four parasite-host tandem cultures (*Chytridium* sp./*Navicula* sp., *Rhizophydium* type I/*Nitzschia* sp., *Rhizophydium* type IIa/*Rhizosolenia* sp., *Rhizophydium* type IIb/*Chaetoceros* sp.; Table 1) were used to test the following hypothesis: (1) abiotic environmental stressors such as temperature, salinity or day length affect parasite-host recognition indirectly by variation of the biochemical composition of the host-diatoms during the acclimatization/adaptation process to the stressor, including the accumulation of compatible solutes; and (2) diatom hosts are able to defend themselves against the onslaught of chytrid zoospores by release of defense molecules (e.g., phenols, flavonoids) into the surrounding medium. Hellendahl staining jars divided in two compartments by nylon filters (mesh opening 5 μm) were utilized in combination with different standard solutions and aqueous host-extracts in order to test the chemotactic responses of zoospores to different concentrations of potential trigger substances. The standards were tested individually but also as compound class sole mixtures (e.g., comprising all eight carbohydrates). In addition, standards were also utilized to simulate the presence of stressed host diatoms (salinities of 10 and 40, temperature of 20 °C and 24 h photoperiod in combination with UVR). To address the topic regarding the accumulation and release of defense molecules, a non-contact-co-culturing approach was realized by using nylon filters with smaller mesh openings (0.5 μm) in combination with resistant and susceptible diatom host-cultures and phytochemical screening methods. In the final zoospore activity screenings, individual biomass and supernatant extracts gained from the phytochemical study were used to test whether or not the infection process was inhibited. In each case the presence (susceptibility) or absence (resistance) of sporangia attached to diatom-hosts was confirmed by fluorescence microscopy, using Calcofluor White for visualization.

## 2. Results

### 2.1. Chemotactic Responses (CR) of Zoospores

**Figure 1:**
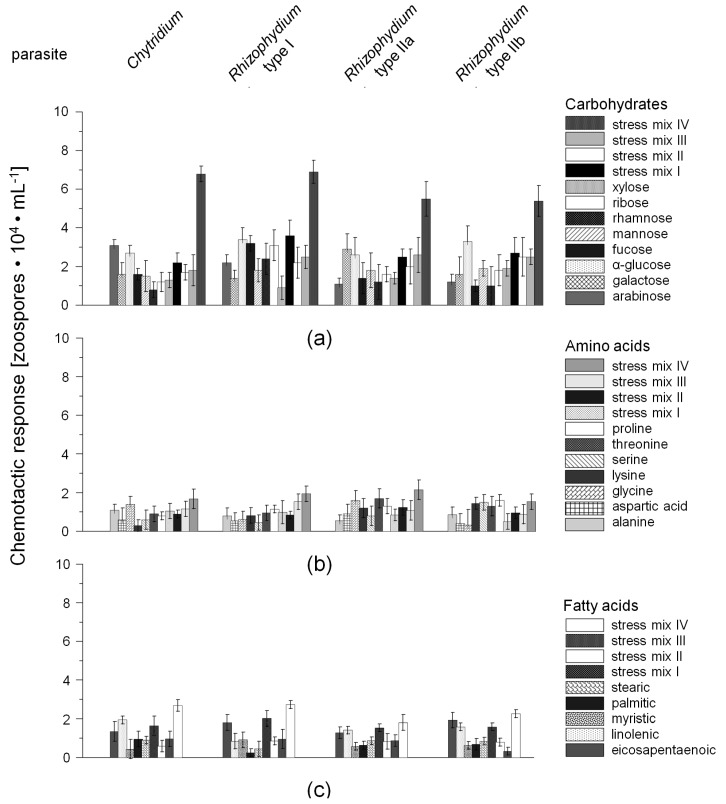

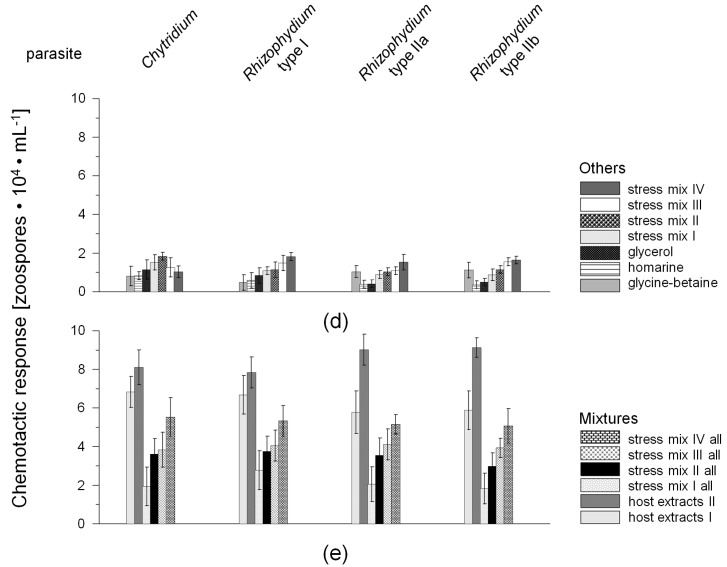
Chemotactic response (CR) of the four chytrid types (*Chytridium* sp., *Rhizophydium* sp. type I and type II a and b) to different attractants tested as individual standards and mixtures: (**a**) carbohydrates; (**b**) amino acids; (**c**) fatty acids; (**d**) others (quaternary ammonium compounds and polyol glycerol); and (**e**) mixtures of standards and host extracts obtained from the specific diatom host cultures grown under standard culture conditions (10 °C, 12:12 h light:dark (L:D), 50 μmol·photons·m^‒2^·s^‒1^; host extract I) and a 24 h light regime, including 6 h UV exposure (host extract II). Furthermore, mixtures simulating the presence of stressed diatoms hosts were also tested (mix I: salinity of 10; mix II: salinity of 40; mix III: temperature of 20 °C and mix IV: 24 light + UVR). The CR values were calculated as described in Section 4.4.1.1, including the data given in Table 3 The individual concentrations of each standard and mixture are given in Table 2.

Overall 43 solutions with standard chemicals were used to test the first hypothesis, comprising individual and mixed standards of 22 compounds (Table 2. In addition, aqueous extracts of the four susceptible host-diatoms (*Navicula* Bory, *Nitzschia* Hassall, *Rhizosolenia* Brightwell, *Chaetoceros* Ehrenberg) grown under standard culture conditions (10 °C, 12:12 h light:dark (L:D), 50 μmol·photons·m^‒2^·s^‒1^) and 24 h light regime with additional UVR exposure, were also tested. All parasite-host experiments were carried out at 10 °C, 16:8 h light:dark (L:D) regime and at a photon fluence rate of 40 μmol·photons·m^‒2^·s^‒1^. Each of the tested solutions depicted gave positive chemotaxis responses of the zoospores, varying only in the strength of attraction (Figure 1). In detail, the filters pre-soaked with whole-cell extracts of the light-stressed hosts attracted the highest numbers of zoospores (CR up to 86%, host extract II, Figure 1e), followed by whole-cell extracts of diatom hosts grown under standard culture conditions (up to 78%, host extract I; Figure 1e), the combined carbohydrate standard solution (CR up to 76%, mix IV, Figure 1a), and the overall standard mixture simulating the presence of light-stressed host-diatoms (mix IV, Figure 1e). From the tested individual carbohydrate standards, α-glucose attracted the highest number of zoospores of all tested chytrid types, varying from 27% to 34% for *Chytridium* sp. and *Rhizophydium* sp. type I, respectively (Figure 1a). In addition, significant differences in the attraction of *Rhizophydium* type IIa and *Chytridium* sp. zoospores were found for galactose and ribose (ANOVA_*Rhizophydium* type IIa vs galactose_ *F*_1,11_ = 30.1, ANOVA_*Chytridium* sp. vs. ribose_ *F*_1,12_ = 29.2, *p* < 0.0001). In contrast, almost all tested individual amino and fatty acid standards, were found to be weak triggers for zoospore chemotaxis, varying only from 5.2%–12.6% of the overall amount of zoospores in the chambers (*p* < 0.05).

### 2.2. Phytochemical Screening

**Figure 2:**
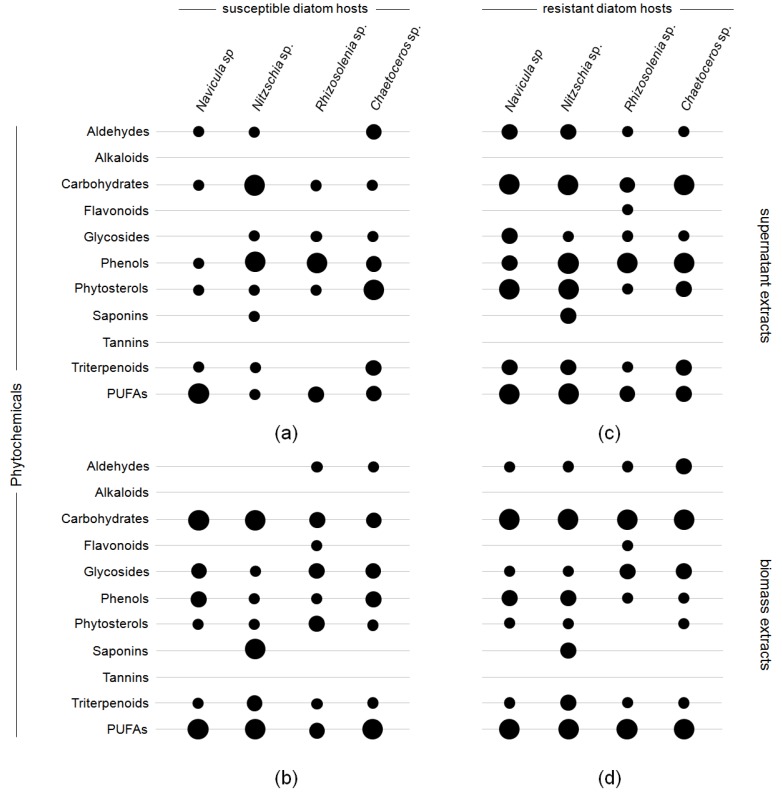
Results of the phytochemical screening of infection susceptible (**a**,**b**) and unsusceptible diatom hosts ((**c**,**d**) *Navicula* sp., *Nitzschia* sp., *Rhizosolenia* sp. and *Chaetoceros* sp.) in a non-contact-co-culturing approach using parasite-host tandem cultures (*Chytridium* sp. vs. *Navicula* sp., *Rhizophydium* sp. type I vs. *Nitzschia* sp. vs. *Rhizophydium* sp. type IIa vs. *Rhizosolenia* sp. and *Rhizophydium* sp. type IIb vs. *Chaetoceros* sp.) as trigger for the accumulation of potential defense molecules. Considered were aldehydes, alkaloids, carbohydrates, flavonoids, glycosides, phenols, phytosterols, saponins, tannins, triterpenoids, and PUFAs (pooled evaporated ethanolic, methanolic, and *n*-hexanic extracts re-dissolved in 100 mL aqueous EtOH (40%); *n* = 6), distinguishing weak (small circles), middle (medium circles), and strong colorimetric responses (large circles) with the exception of the test for saponins. (**a**,**c**) describes the effects of supernatant extracts; (**b**,**d**) those of biomass extracts. For details of the conducted tests, see Table 4.

Testing pooled ethanolic, methanolic, and *n*-hexanic extracts of resistant and susceptible diatom taxa for the presence of potential defense compounds (e.g., polyunsaturated fatty acids (PUFAs), carbohydrates, glycosides, phenols, phytosterols, and triterpenoids) yielded only minor differences between the resistant and susceptible diatom taxa (Figure 2). Particularly, aldehydes were more abundantly found in biomass and supernatant extracts of resistant diatom taxa (Figure 2c,d), being significant in biomass extracts of *Chaetoceros* sp. and supernatant extracts of *Navicula* sp. and *Nitzschia* sp. (ANOVA_*Chaetoceros* sp._ *F*_1,12_ = 30.9, ANOVA_*Navicula* sp._ 29.3, and ANOVA_*Nitzschia* sp._ 31.4, *p* < 0.0001). The accumulation of carbohydrates and PUFAs was also significant in extracts of unsusceptible *Navicula* and *Nitzschia* biomass and supernatant extracts (*p* < 0.05). While alkaloids and tannins were in general not present in either biomass or supernatant extracts, the froth tests for saponins was positive for biomass and supernatant extracts of *Nitzschia* sp. with no significant concentration difference between the susceptible and the resistant strain (*p* > 0.05). In addition, positive colorimetric reactions in the Alkaline Reagent Test indicated the presence of a flavonoid in the biomass extracts of susceptible and resistant *Rhizosolenia* sp. In contrast, the test gave a positive test result only for the resistant Rhizosolenia also in supernatant extract.

### 2.3. Zoospore Activity Screening

**Figure 3:**
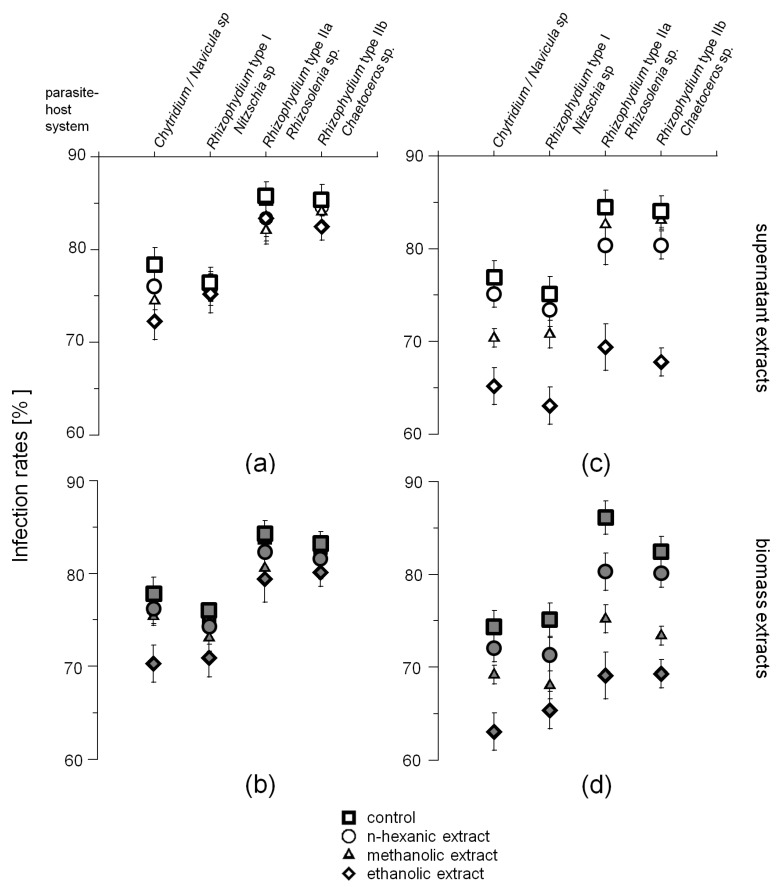
Results of the zoospore activity screening, using supernatant (**a**,**c**) and biomass (**b**,**d**) extracts obtained from the phytochemical screening conducted according to the methods given in Table 4 Depicted are the infection rates of the four diatom hosts (*Navicula* sp., *Nitzschia* sp., *Rhizosolenia* sp. and *Chaetoceros* sp.) and their chytrid parasites exposed to *n*-hexanic, methanolic and ethanolic extracts of infection susceptible (**a**,**b**) and unsusceptible (**c**,**d**) diatom hosts grown under standard culture conditions.

**Figure 4:**
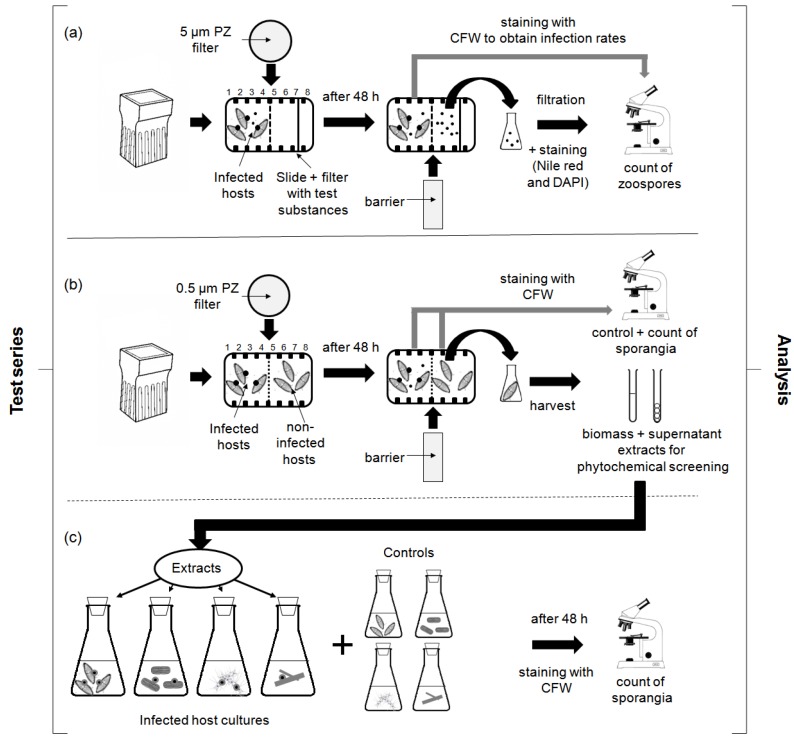
Illustration of the methods used for the different experiments: (**a**) chemotaxis experiment; (**b**) screening for defense molecules, and (**c**) activity screening, using the extracts obtained from the non-contact-co-culturing approach in combination with the infected and non-infected diatom host cultures.

The infection rates of the four diatom hosts (*Navicula* sp., *Nitzschia* sp., *Rhizosolenia* sp., and *Chaetoceros* sp.) exposed to 2 mL *n*-hexanic, methanolic, and ethanolic extracts re-dissolved in 100 mL aqueous EtOH (40%), obtained from the phytochemical screening showed distinct differences between susceptible and resistant diatom hosts (Figure 3). In the negative controls, using 2 mL medium and aqueous EtOH (40%), respectively, no inhibition of growth and infections was observed, resulting in an infection prevalence of 98.5% ± 0.5% (*n* = 9). Infection rates of the experiments ranged from 74% to 86%, being the lowest for *Nitzschia* sp. infected by *Rhizophydium* type I (with the exception of the assay depicted in Figure 4d) and the highest for *Rhizosolenia* sp. infected by *Rhizophydium* type IIa. Inhibitive effects on the infections were predominantly caused by ethanolic biomass and supernatant extracts of the resistant diatom strains, being significant for *Rhizosolenia* and *Chaetoceros* infected by *Rhizophydium* type II a and b, respectively (ANOVA *F*_1,12_ = 32.4, *p* < 0.0001, 21.3% and 24.6%, Figure 3c,d). In addition, methanolic extracts of the resistant diatom strains were also found to inhibit infections by the parasites with significant differences between biomass and supernatant extract (*p* < 0.05).

## 3. Discussion

### 3.1. Critical Remarks

Although in a pre-trial the distribution of a staining colorant in the Hellendahl staining jars under experimental conditions was tested, the diffusion properties of each compound used in the chemotaxis and defense screening experiments were not characterized individually. In general, diffusion is highly related to physical–chemical properties of compounds and it is defined as the random thermal movement of molecules in a solution, and thus diffusion may only cause a net transport of molecules in the presence of a concentration gradient [48].

The diffusion rate is related to the diffusion coefficient of a solute, a constant related to the properties of a given molecule in a given solvent [49]. Usually the coefficients are defined for solutions in double-distilled water. In our case two additional factors have to be considered: (1) All experiments were conducted in seawater, which represents a chemically complex matrix; (2) chambers included microorganisms. The presence of microorganisms might lead to changes of the net transport of molecules in the solute, because of uncontrolled up-take and exudation. Thus the results of the numerous assays conducted for the present study, provide only an indication in which direction further research for attractants and defense molecules might go—they cannot contribute to the mechanistic understanding of chemotaxis and defense in the tested species in general.

### 3.2. Chemotaxis of Chytrid Zoospores

Chemotaxis is an important virulence factor for many pathogenic microorganisms (e.g., [50]). Zoosporic fungi rely on the presence of one or more flagella to aid in their ability to swim through liquid environments [51]. In addition, several studies indicate that some species of motile, pathogenic microorganisms display positive migration toward suitable host and nutrient substrates, indicating that their ability to infect a host involves not only motility but also chemotaxis [52].

In the present study, all tested standards gave positive chemotactic responses, but the strength of the responses, reflected in the number of attracted zoospores, varied considerably between individual and mixed standards tested during the experiments. In almost all cases, using individual standards, the number of attracted zoospores was 36%–68% lower compared to mixed standards and whole cell extracts of the hosts. Similar to the findings of the present study, Muehlstein et al. [22], investigating the marine chytrid *Rhizophydium littoreum*, a parasite (but not pathogen) of marine green algae, reported that individual standards of carbohydrates attracted more zoospores than those of amino or fatty acids. The authors related this observation to the presence of carbohydrate-specific sensory receptors. In addition, it was found by Muehlstein et al. [22] that for example particular amino acids such as proline supported the growth of the parasite, but did not attract zoospores during their experiments at all. The authors stated that in several cases chemicals which supported growth were not necessarily positive chemo-attractants, and conversely, chemo-attractants did not necessarily act as sources of nutritional carbon or nitrogen. Although the pathogens in the present study could not be further isolated, or at least could not be stabilized in culture over a longer time period, the overall results suggest that at least one sensory receptor must be present for such a selective swimming behavior as recorded during the current experiments. Furthermore, using the described individual standards, the zoospores were either dead or encysted after 72 h of incubation time. This latter process was only reversible in the presence of a suitable living host, indicating that even mixed solutions did not reflect the complete nutritional needs of the tested pathogens. In a similar case, the pathogen *Chytridium polysiphoniae* was found to preferentially infect young, fast-growing cells of the filamentous brown alga *Pylaiella littoralis* [53,54]. In light of the results presented here, it is tempting to hypothesize that pathogen zoospores were preferentially attracted to the fast-growing cells by photosynthesis-derived carbohydrate exudates.

#### 3.2.1. Role of Environmental Factors

One of the basic assumptions in parasitology is that parasites, and in particular pathogens, vary in virulence and infection transmission due to manifold differences in the host, the pathogen, and the environment. Particularly the biochemical composition of the host, and thus the nutritional value for the parasite, can vary with environmental conditions (e.g., [55]). The results of the present study not only indicate that the tested chytrids might utilize chemotaxis for the purpose of finding potential host diatoms. Moreover, the results suggest that the pathogens, or rather their infective propagules, the zoospores, seem to be able to distinguish between different biochemical compositions of the potential host diatoms and respond more to light-stressed host cells. In this context, the accumulation of organic osmolytes by marine diatoms as relatively rapid reaction (6–24 h) to multiple changes in abiotic environmental conditions has been hypothesized in the present study to affect positively the chemotactic response of the chytrid zoospores. Besides free amino acids, such as proline, several other organic osmolytes have been detected in marine and estuarine diatoms such as glycine-betaine [56], homarine [56,57], cyclohexanetetrol [58,59], glycerol [56], and mannose [60]. While the latter two are direct products of photosynthesis, like sugars (e.g., sucrose, trehalose), polyols (e.g., glycerol, mannitol, sorbitol) and heterosides (e.g., floridoside, isofloridoside), the former ones are not directly derived from photosynthesis [56,57,58,59]. Most of these compounds have been mainly described in relation to salinity stress (e.g., [31,32,56]), but were also found to play major roles in relation to other stressors (e.g., [33,34,35]). In relation to the present study, the hypothesis of a major role of most of these organic osmolytes as chemo attractants for zoospores has to be rejected. Although stronger positive chemotactic responses were recorded for the mixtures simulating temperature and salinity stressed hosts, light-stressed diatoms seem to be the most important item in the attraction process.

#### 3.2.2. Chemotaxis versus Other Factors or Attractants

Besides the chemotactic responses of the zoospores to the attractants, two phenomena occurred during the conducted experiments. The first one was that in all test assays approximately 10%–20% of the zoospores released by the mature sporangia were still found on the left side of the chambers, remaining with the already infected diatom hosts (the infection rate was around 99% after 24 h in this test series). The second phenomenon was that in almost all assays the zoospores partly aggregated on the surface of the liquid phase on both sides of the test chambers. Similar effects linked to light exposure were also described for *Rhizophydium planktonicum* in Canter and Jaworski [61]. Although comparable literature about chytridiomycosis of marine diatoms has not been published yet, examples can be found in the research focusing on other zoosporic parasites such as oomycetes which are well known as plant and fish pathogens (e.g., [62]). For such pathogens, bioconvection (=directional motion of large numbers of small organisms in a fluid) and chemotaxis are both described as common concentrative phenomena [63]. In theory each of these processes could potentially lead to zoospore populations in a natural environment becoming more localized, hence increasing infection pressure at spatially distinct sites [63]. In this context, it was shown that each of these mechanisms has a specific role to play, in which bioconvection causes the rapid auto-aggregation of zoospores into plumes that have a maximum density at the surface of a suspension. It has been shown that only when these aggregates have formed, does chemotaxis seem to be sufficiently strong to drag these together to form super-aggregates over a much longer time scale [63]. Although such super-aggregates were not observed after the incubation time in the present study, it is highly likely that chytrid zoospores gather in a similar way as described above in order to maximize their potential to localize a susceptible host and maybe also to guarantee that at least one of the infective propagules might be able to overcome the potential risks of being grazed in a natural environment (swarm principle).

Regarding the phenomenon of the zoospores remaining close to the already infected diatom hosts, one idea to explain this is that electrical fields might have an additional influence as shown for the plant pathogenic oomycete *Phytophthora palmivora* [62]. In general, eukaryotic organisms are known to generate exogenous voltages and circulating endogenous ionic currents, due to the spatial segregation of ion pumps and ion channels in the cell membrane at distinct domains, or poles of cells. For plant pathogenic oomycetes it has been shown that chemotaxis alone did not explain the conundrum that marked spatial differences exist in the sites of accumulation of zoospores at the root surface (e.g., [62,63,64]). Beyond that, the data of van West et al. [62] suggest that electrical signals can augment or override chemical ones in mediating short-range tactic responses of oomycete zoospores at root surfaces. In principle, the observed remaining of chytrid zoospores with almost dead and infected hosts could be related to such an interaction but needs to be further investigated.

### 3.3. Potential Host-Defense Molecules and Infection Prevalence

In diatoms, recent reports have clearly demonstrated that chemical defense against grazing also relies on the products of fatty-acid oxidation. For the planktonic diatoms *Asterionella* and *Thalassiossira* it has been shown that only seconds after the cells were mechanically wounded, an enzymatic mechanism produced fatty acid derived metabolites, resulting in the release of α,β,γ,δ-unsaturated aldehydes (e.g., [25,26]). Besides these examples of chemical defense in marine diatoms, several of the compound classes detected in the biomass and supernatant extracts in the present study, using phytochemical screening methods, have been tested positive as antimicrobials (e.g., flavonoids [65]). To which extent these compounds effectively contribute to the defense of diatoms against the onslaught of chytrid zoospores is still unknown and has to be tested in further single compound studies. In addition, it has to be stated clearly that the detected compounds might also originate from the pathogens since an exchange of molecules in both directions was possible during the conducted experiments. One of the most interesting findings from the screenings is the activity of the ethanolic extract (and to a lesser extent also the methanolic one) against the zoospores and thereby the reduction of infections. Also in this case further investigations are needed and more definitive studies must be conducted to determine the precise mechanism of attraction of zoospores to each of the chemicals tested. The limiting factor of the methods used in the present study is clearly the small volume which makes further metabolomic approaches almost impossible, but on the other hand these small volumes were necessary due to the restricted swimming distances of the zoospores and thus will need further technical developments to overcome these limitations.

## 4. Materials and Methods

### 4.1. Chemicals

If not otherwise mentioned, all chemicals used in this study were of the highest purity from Sigma/Aldrich Chemical Co. (St. Louis, MA, USA).

### 4.2. Organisms

#### 4.2.1. Isolation and Maintenance Prior to Experiments

The four marine chytrid-diatom pairs, which were used in the test series, were isolated from three coastal areas in north-west Iceland in 2015 and maintained as dual cultures, because all attempts to further isolate the parasite failed [66]. *Navicula* and *Nitzschia* and their pathogens were isolated by the spreading plating method, using sediment samples obtained during a short-term monitoring conducted at the Isafjördur and Hrútafjördur (65°54′40.46″ N, 22°21′37.99″ W and 65°09′13.40″ N, 21°05′18.47″ W, respectively) in May and September 2015, respectively, whereas *Rhizosolenia* and *Chaetoceros* and their pathogens were isolated using the *serial-dilution* technique from enriched phytoplankton sub-cultures obtained from a long-term monitoring program in the Skagaströnd area in June 2015 (65°49′36.60″ N, 20°20′17.37″ W). In each case individual cells were isolated with a micropipette into microwells containing 1.5 mL of the culture medium f/2-Si ([67], with and without agar). A mixture of artificial seawater salt Tropic Marin^®^ (GmbH Aquarientechnik, Wartenberg, Germany) and sterile-filtrated natural seawater (50% *v*/*v*) at a salinity of 30 was used for the isolation of host and parasite pairs. In parallel, non-infected *Navicula*, *Nitzschia*, *Rhizosolenia* and *Chaetoceros* were collected and cultivated as monoclonal cultures. From each host species only two of the monoclonal cultures were cultivated in high densities as host supply for the pathogens. The non-infected diatoms were purified from bacterial contaminants by transfer of the cells into microwells containing 0.5 mL f/2-medium, 5 μg·mL^−1^ tetracycline and 5 μg·mL^−1^ kanamycin. In the case of *Navicula* sp. and *Nitzschia* sp. 1.5% agar was used. The absence of bacterial contaminants was verified by epifluorescence microscopy using the dyes 4′,6-diamidino-2-phenylindol (DAPI). Each isolate was maintained in 250 mL batch cultures under sterile conditions at 10 °C, 12:12 h light:dark (L:D) regime and at an irradiance of 50 μmol·photons·m^‒2^·s^‒1^ using Master TL-D 18W/840 light (Phillips, Hamburg, Germany). Three weeks prior to experiments isolates and parasite-host tandem cultures were scaled up to 1000 mL culture volume.

#### 4.2.2. Species Identification

For diatom species determination, cleaned valves of each cultured strain were prepared according to the method of Schrader [68], embedded in Naphrax (refractive index 1.74, Northern Biological Supplies Ltd., Ipswich, UK) and examined under a light microscope (Olympus BX51, Olympus Europe SE & Co. KG, Hamburg, Germany) at 1000× magnification. For species identification the literature referenced in Scholz & Einarsson [69] was used.

### 4.3. Experimental Designs

All experiments were conducted in triplicates over a time period of 24 h, utilizing autoclaved Hellendahl glass staining jars (rectangular, 90.2 mm high, 44.5 mm width, 58.8 mm length, containing eight slide holders; Isolab, Wertheim, Germany). As barriers between infected diatom hosts (host-parasite dual cultures) and the different test approaches, nylon filters (Spectrum™ Spectra Mesh™; Fisher Scientific, Schwerte, Germany; 47 mm in diameter) with different mesh openings were used (5 μm for the zoospore experiment and 0.5 μm in the non-contact-co-culturing approach, Figure 4). The nylon filters were adjusted in the middle holder of each jar and the chambers were filled with a total volume of 60 mL. Each experiment started by adding 30 mL of infected and monoclonal (uninfected) test cultures into the first chamber compartment, containing already 30 mL fresh f/2 culture medium. For closing the jars and in order to prevent anoxic conditions, autoclaved cotton wool was used. The experiments were carried out at 10 °C, 16:8 h light:dark (L:D) regime and at an irradiance of 40 μmol·photons·m^‒2^·s^‒1^ using Master TL-D 18W/840 light (Phillips, Hamburg, Germany) in an incubator (Innova 42R, Eppendorf New Brunswick Co., Inc., Hamburg, Germany) without shaking the test approaches. The position of replicate jars was randomly changed every other day to eliminate any location effect due to minor changes in external conditions. The diffusion properties in the jars were tested once initially, using 10 μL of Methyl blue (C_37_H_27_N_3_Na_2_O_9_S_3_; 2% aqueous solution). The test was conducted by placing the solution into the middle of the right side of a jar, which was already filled with f/2 medium, and observing the distribution of the dispersion for 72 h under experimental conditions. The test was conducted in triplicate and the Methyl blue solution was evenly distributed on the right side of each jar after 18.5 ± 0.5 h and in the whole chamber after 48.5 ± 1.5 h.

#### 4.3.1. Chemotaxis Experiments

Cellulose filters (Whatman^®^ diameter 47 mm, grade 1) were pre-soaked, using standards of eight carbohydrates, seven amino acids, five fatty acids, and three compounds known as compatible solutes (e.g., homarine = *N*-methyl picolinic acid and glycine-betaine) in individual and mixed solutions (Table 2). In addition, aqueous extracts of monoclonal host-diatoms grown under standard conditions as described in Section 4.2.1 (control) and 24:0 light:dark cycle with supplemented UVR were also tested, following the protocols given in Section 4.3.2. The specific growth rates reached during the prior-experiments are given in Table S1 (Supplementary Materials). The specific growth rates are presented in Table 4. The standards and evaporated host-extracts were solved in sterile chemotaxis buffer (pH 7.35; 13.78 g of NaCl, 0.013 g of K_2_HPO_4_, 0.027 g of KH_2_PO_4_, 2.60 g of MgSO_4_·7H_2_O, and 0.525 g of CaCl_2_·2H_2_O per liter of H_2_O) and the concentration of each acid and carbohydrate was adjusted according to its natural composition in marine diatoms exposed to standard and selected environmental stressors, respectively (osmotic, temperature and light (UV) stress; [33,34,35]; Table 4). The pre-soaked filters were wrapped around glass slides (BRAND^®^ microscope slide, size 76 × 26 × 1 mm) and adjusted in the seventh holder of the Hellendahl glass staining jars (Figure 4a). Three controls were used in the tests: (1) cellulose filters pre-soaked with sterile chemotaxis buffer (without standard solution); (2) cellulose filters pre-soaked with f/2 medium; and (3) glass slides without filters. The 5 mL suspension with the infected parasite-host tandem cultures was adjusted to contain 0.8 × 10^8^ to 6.3 × 10^8^ zoospores per ml (cell density: 7.9–9.8 × 10^6^ cells per mL; infection rate of the infected cultures: 95% ± 5%). At the end of the incubation period glass slides (size 76 mm × 34.5 mm × 1 mm) were inserted in the fifth holder of each staining jar, the liquid phase of each test approach was removed by suction with a plastic tube, collected in Erlenmeyer flasks and analyzed as described in Section 4.4.1.1.

#### 4.3.2. Screening for Defense Molecules (Non-Contact-Co-Culturing Approach)

Monoclonal cultures from each diatom host-species, which were found to be resistant in prior experiments [66], were used for the present screening. In addition, monoclonal cultures of susceptible diatom hosts were also used in the experiments. In each approach the non-infected host cultures were added to the right side of the staining jars (cell density: 9.1–9.8 × 10^6^ cells per mL) whereas on the left side parasite-host tandem cultures were placed (cell density: 6.8–9.2 × 10^6^ cells per mL; infection rate of the infected cultures: 50% ± 2%). In this non-contact-co-culturing approach, nylon filters (Spectrum™ Spectra Mesh™; Fisher Scientific, Schwerte, Germany; 47 mm in diameter) with a mesh opening of 0.5 μm were used (Figure 4b). After incubation time, the triplicates were pooled, the biomass was harvested by centrifugation (15 min at 6000 rpm), and the cell free supernatants were decanted, filtered (0.45 μm, Whatman GF/C, Sigma/Aldrich Chemical Co. St. Louis, MA, USA), and evaporated (30 °C; Rotavapor R-114, B-480, Büchi, Flawil, Switzerland). The biomass of each test was weighed, equalized to a final weight of 1.5 g fresh weight and sonicated 3 times for 2 min under cooling conditions (Branson 2800, Emerson Electric Co., Ferguson, MO, USA).

The homogenized biomass was then extracted in three different solvents (each 1 h) in an all-glass filtration chamber, using 50 mL ethanol (99.5%; for flavonoids, alkaloids, phenols, tannins, saponins, etc.), 50 mL methanol (80%; for aldehydes and sterols) and 50 mL *n*-hexane (PUFAs), following different methods referenced in Table 4 With the exception of the sonication, the same extraction procedure was conducted for the evaporated supernatant extracts. The residues from all extractions were evaporated and re-dissolved in 100 mL aqueous EtOH (40%), filtered (Whatman GF/C, 47 mm), and used for the activity (Section 4.3.3) and phytochemical screening (Section 4.4.2).

#### 4.3.3. Zoospore Activity Screening

Activity screening was conducted with liquid culture assays (LCA) in 300 mL Erlenmeyer flasks (Isolab, Wertheim, Germany; culture volume 150 mL), using *n*-hexanic, methanolic, and ethanolic biomass and supernatant extracts of susceptible and unsusceptible host-diatoms obtained from the screening assays described in Section 4.3.2 (re-dissolved in aqueous EtOH (40%); test volume each 2 mL) in combination with parasite-host tandem cultures (*Chytridium* sp./*Navicula* sp., *Rhizophydium* type I/*Nitzschia* sp., *Rhizophydium* type IIa/*Rhizosolenia* sp., *Rhizophydium* type IIb/*Chaetoceros* sp.) as well as resistant host cultures of *Navicula* sp., *Nitzschia* sp., *Rhizosolenia* sp. and *Chaetoceros* sp. (Figure 4c). The tests were accompanied by two controls. The first control was used to estimate infection rates without any external influence. In addition, a negative control for each test was included, which consisted of species exposed to aqueous EtOH (40%) instead of the extracts in the same volume as described above.

### 4.4. Analysis

#### 4.4.1. Microscopy

##### 4.4.1.1. Number of Zoospores

After the chemotaxis experiments, the liquid fractions in the Erlenmeyer flasks were filtered (Whatman Nuclepore polycarbonate black filters, pore size, 0.2 μm), stained using Nile Red and DAPI and the number of zoospores determined using epifluorescence microscopy (Olympus BX51, equipped with a BX-RFA reflected fluorescence system, Hamburg, Germany). For quantification of the zoospore responses to the potential chemoattractant, the chemotactic ratio was determined by the following formula:

Chemotactic response (CR) = mean spore numbers of assay − mean spore numbers of control
(1)

The spore numbers are given in Table 3 and CR was calculated for each tandem culture and control separately and given in Figure 1 as mean value ± standard deviation (SD).

##### 4.4.1.2. Infection Prevalence

The infection rates (prevalence) in all experiments were determined by counting the test cultures (3 × 100 mL) under a microscope (1000× magnification) using Fluka Analytical Calcofluor White staining (18909-100ML-F) in combination with UV-light, considering in each case only the sporangia attached to host cells in formaldehyde fixed samples (2% *v*/*v*). The percentage of infected cells was calculated by dividing the number of infected cells by the total number of host cells. In addition, the mean number of chytrids per cell (host) was also calculated, by dividing the total number of parasites attached to algal cells by the total number of host cells, to normalize the cell density among treatments. This value is referred to as the mean intensity of infection [76], reflecting the number of pathogens that succeed in attaching to their host.

#### 4.4.2. Phytochemical Screening

Phytochemical screening was used as a pre-test system to ascertain distinct compound classes without detailed structural assignment of the chemicals [71,77]. This qualitative phytochemical analysis was performed for the presence of alkaloids, tannins, saponins, flavonoids, phenolic compounds, aldehydes, sterols, terpenes, and PUFAs, utilizing the pooled extracts and the extraction solvents used as controls. In addition, concentrations were determined for each compound class using the standards und methods referenced in Table 4. The colorimetric reactions were classified into three categories (with the exception of saponins for which only the presence or absence was noted).

#### 4.4.3. Statistical Analysis

Coefficient of variation (CV) for the positive and negative controls of each test was calculated to ascertain reproducibility. Data are expressed as the arithmetic mean ± standard error of the mean (SEM). To assess variability of test results within the different species (parasite-host tandem cultures, chytrid zoospores, and host diatoms), analyses of variance (one way ANOVA) were performed on logarithmically transformed data to adhere to normality. Differences were expressed as percentage values and mean values among treatments were compared by the Duncan’s multiple range test at the 5% (*p* = 0.05) level of significance as a post-hoc test. All tests were performed with the program XLSTAT 2011, Version 2011.2.08 Addinsoft.

## Figures and Tables

**Table 1:** Description of the chytrid strains used in this study and maintained in parasite-host tandem cultures, including strain labels of susceptible and resistant diatom species ^1^. For identification of chytrids the studies of Sparrow [16], Johnson & Sparrow [46], and Letcher & Powell [47] were used.

Host Species (Author)	Parasite	Description of the Parasites
*Navicula* Bory NavHrú1542S NavHrú1523R	*Chytridium* type I	Sporangium on a stalk (extrametrical, up to 15.8 μm long), ovate or ellipsoid (also spherical was observed), around 16 μm long by 10–16 μm wide. Similarities with *Chytridium* sp.
*Nitzschia* Hassall NitzIsa15101S NitzIsa15106R	*Rhizophydium* type I	Sporangium: sessile, ovoid or spherical (globose), 17–35 μm high, 17–40 μm diameter; wall thin and smooth, colorless, double-contoured, 1–1.5 μm thick, with a broad apical or sub apical papilla. Similarities with *Rhizophydium* Schenk
*Rhizosolenia* Brightwell RhizSka1503S RhizSka1512R *Chaetoceros* Ehrenberg ChaeSka1517S ChaeSka1511R	*Rhizophydium* type II	Sporangium: sessile, ovoid, 5–11 μm high by 5–9 μm in diameter, wall smooth, colorless. Similarities with *Rhizophydium* Schenk

^1^ Strains are maintained at the indoor culture collection at BioPol ehf.

**Table 2:** Concentrations of standards used in the chemotaxis experiments. Included are specifications of mixtures utilized to simulate the presence of stressed host diatoms (Mix I: salinity of 10; Mix II: salinity of 40; Mix III: temperature of 20 °C and Mix IV: 24 light + UVR, according to data given in [33,34,35]).

Compounds (g·L^−1^)	Concentrations				
	Individual	Mix I	Mix II	Mix III	Mix IV
Carbohydrates					
arabinose	1.1 × 10^−2^	1.3 × 10^−2^	0.9 × 10^−2^	0.8 × 10^−2^	1.5 × 10^−2^
galactose	1.4 × 10^−2^	1.2 × 10^−2^	1.5 × 10^−2^	2.0 × 10^−2^	4.5 × 10^−2^
α-glucose	5.5 × 10^−1^	4.3 × 10^−1^	4.6 × 10^−1^	3.9 × 10^−1^	3.7 × 10^−1^
fucose	2.6 × 10^−2^	2.3 × 10^−2^	2.1 × 10^−2^	1.5 × 10^−2^	1.8 × 10^−2^
mannose	3.3 × 10^−2^	4.3 × 10^−2^	4.6 × 10^−1^	2.5 × 10^−1^	3.5 × 10^−1^
rhamnose	1.5 × 10^−1^	2.6 × 10^−1^	2.4 × 10^−1^	2.9 × 10^−1^	2.8 × 10^−1^
ribose	2.8 × 10^−1^	2.5 × 10^−1^	2.7 × 10^−1^	3.4 × 10^−1^	2.4 × 10^−1^
xylose	1.9 × 10^−2^	1.3 × 10^−2^	1.5 × 10^−2^	2.0 × 10^−2^	3.5 × 10^−2^
Amino acids					
alanine	3.5 × 10^−2^	8.5 × 10^−2^	1.9 × 10^−2^	0.7 × 10^−3^	0.5 × 10^−3^
aspartic acid	8.2 × 10^−1^	4.3 × 10^−1^	1.2 × 10^−2^	8.2 × 10^−2^	1.5 × 10^−1^
glycine	6.5 × 10^−3^	8.4 × 10^−3^	2.3 × 10^−3^	1.1 × 10^−3^	1.0 × 10^−3^
lysine	4.2 × 10^−3^	2.7 × 10^−2^	0.5 × 10^−2^	0.5 × 10^−2^	3.4 × 10^−3^
serine	5.3 × 10^−2^	8.1 × 10^−2^	8.3 × 10^−2^	1.2 × 10^−2^	0.6 × 10^−2^
threonine	3.5 × 10^−2^	6.4 × 10^−3^	3.9 × 10^−3^	8.8 × 10^−2^	8.2 × 10^−2^
proline	4.2 × 10^−3^	50.1 × 10^−1^	20.9 × 10^−1^	15.3 × 10^−1^	56.0 × 10^−1^
Fatty acids					
eicosapentaenoic (20:5)	3.8 × 10^−2^	4.5 × 10^−2^	4.9 × 10^−1^	6.8 × 10^−1^	8.3 × 10^−2^
linolenic (18:2)	1.1 × 10^−3^	9.8 × 10^−2^	9.3 × 10^−2^	6.4 × 10^−2^	8.1 × 10^−2^
myristic (14:0)	5.2 × 10^−4^	5.3 × 10^−3^	5.5 × 10^−4^	4.9 × 10^−4^	2.3 × 10^−4^
palmitic (16:0)	5.1 × 10^−4^	5.6 × 10^−3^	5.0 × 10^−4^	6.7 × 10^−4^	1.5 × 10^−4^
stearic (18:0)	5.9 × 10^−4^	6.2 × 10^−3^	6.3 × 10^−3^	3.4 × 10^−4^	3.2 × 10^−4^
Others ^1^					
glycine-betaine	1.1 × 10^−4^	1.0 × 10^−2^	3.9 × 10^−3^	1.1 × 10^−2^	4.3 × 10^−2^
homarine	0.8 × 10^−4^	1.7 × 10^−2^	2.3 × 10^−3^	3.0 × 10^−2^	3.3 × 10^−2^
glycerol	1.2 × 10^−4^	2.7 × 10^−2^	1.6 × 10^−3^	4.1 × 10^−2^	5.1 × 10^−2^

^1^ Quaternary ammonium compounds such as glycine–betaine and homarine (N-methyl picolinic acid) and the polyol glycerol.

**Table 3:** Numbers of zoospores (10^4^ mL^−1^) of the four chytrid-diatom tandem cultures in the control approaches used during the chemotaxis experiments: (1) cellulose filters pre-soaked with sterile chemotaxis buffer (without standard solution); (2) cellulose filters pre-soaked with f/2 medium; and (3) glass slides without filters). Results are means from triplicate approaches and countings (*n* = 9).

Chytrid-Diatom Pair	Number of Zoospores		
	Control 1	Control 2	Control 3
*Chytridium* sp./*Navicula* sp.	0.052	0.041	0.049
*Rhizophydium* type I/*Nitzschia* sp.	0.083	0.086	0.088
*Rhizophydium* type IIa/*Rhizosolenia* sp.	0.147	0.129	0.141
*Rhizophydium* type IIb/*Chaetoceros* sp.	0.135	0.123	0.139

**Table 4:** Methods used during the phytochemical screening.

Compound Group	Extraction Solvent	Calibration Standard	Method	Reference
Aldehydes	MeOH (80%)	Formaldehyde CH_2_O	Schiff's and Fehling‘s tests *	[70]
Alkaloids	EtOH (99%)	Piperine C_17_H_19_NO_3_	Mayer’s and Wagner’s reagent *	[71]
Carbohydrates	EtOH (99%)	D-glucose C_6_H_12_O_6_	Fehling’s Test	[72]
PUFA	*n*-Hexane	Stearidonic acid C_18_H_28_O_2_	Argentation thin layer chromatography ^1^	[73]
Flavonoids	EtOH (99%)	Quercetin C_15_H_10_O_7_	Alkaline Reagent Test	[72]
Glycosides	EtOH (99%)	Oleandrin C_32_H_48_O_9_	Keller-Killiani Test	[74]
Phenols	EtOH (99%)	Hydroquinone C_6_H_6_O_2_	Folin-Ciocalteu reagent/FeCl_3_	[75]
Phytosterols	MeOH (80%)	Ergosterol C_28_H_44_O	Liebermann-Burchardt test	[72]
Saponins	EtOH (99%)	Saponin S4521	Frothing test	[71]
Tannins	EtOH (99%)	Tannic acid C_76_H_52_O_46_	Gelatine-Saltblock test	[71]
Triterpenoides	EtOH (99%)	18β-Oleanane C_30_H_52_	Salkowski’s Test	[75]

* Only samples that gave positive reactions to both reagents and tests are assumed to contain alkaloids or aldehydes, respectively. ^1^ Methyl esters of PUFAs were separated by argentation chromatography using silver nitrate-impregnated thin-layer chromatography plates. Abbreviations: EtOH: ethanol; MeOH: methanol. PUFA: polyunsaturated fatty acid.

## Supplementary Materials

The following are available online at www.mdpi.com/1660-3397/15/2/26/s1, Table S1: Specific growth rates (µ∙d^1^) ^1^ of the four diatom hosts during prior-experiments in order to obtain biomasses for the present study, using a photoperiod of 24 h light supplemented with UVR ^2^ (6 h∙day^−1^). The experiments were conducted over a period of seven days. Results are means from triplicate approaches and countings (*n* = 9).
