# Supplementary material for: Chytridiomycosis of Marine Diatoms—The Role of Stress Physiology and Resistance in Parasite-Host Recognition and Accumulation of Defense Molecules
Source: Mar Drugs. 2017 Jan 25;15(2):26. doi: 10.3390/md15020026 (PMC5334607; doi:10.3390/md15020026)
Supplement: Supplementary file 1 [file marinedrugs-15-00026-s001.docx]

Supplementary Materials: Chytridiomycosis of Marine Diatoms—The Role of Stress Physiology and Resistance in Parasite-Host Recognition and Accumulation of Defense Molecules

Bettina Scholz, Frithjof C. Küpper, Wim Vyverman, Halldór G. Ólafsson and Ulf Karsten

**Table S1.** Specific growth rates (µ∙d^1^) ^1^ of the four diatom hosts during prior-experiments in order to obtain biomasses for the present study, using a photoperiod of 24 h light supplemented with UVR ^2^
(6 h∙day^−1^). The experiments were conducted over a period of seven days. Results are means from triplicate approaches and countings (*n* = 9).

| **Species** | **Control** | **Photoperiod 24 h + UVR** |
| --- | --- | --- |
| *Navicula* sp. | 0.93 ± 0.04 | 0.51 ± 0.09 |
| *Nitzschia* sp. | 0.41 ± 0.02 | 0.32 ± 0.01 |
| *Rhizosolenia* sp. | 1.22 ± 0.05 | 0.84 ± 0.03 |
| *Chaetoceros* sp. | 1.05 ± 0.01 | 0.96 ± 0.03 |

^1^ The specific growth rate (µ) was calculated with the following equation µ = ln_c1_ − ln_C0_/t_1_ − t_0_; where c_1_ and c_0_ are the number of cells at time t_1_ and t_0_; ^2^ UV-A and UV-B radiation, defined as 280–320 nm, were 4.1 and 0.5 W·m^−2^, respectively, provided by Q-Panel UV-340 fluorescent tubes.
